# Multi-Mobile Agent Trust Framework for Mitigating Internal Attacks and Augmenting RPL Security

**DOI:** 10.3390/s22124539

**Published:** 2022-06-16

**Authors:** Umer Farooq, Muhammad Asim, Noshina Tariq, Thar Baker, Ali Ismail Awad

**Affiliations:** 1 Department of Cyber Security, National University of Computer and Emerging Sciences, Islamabad 44000, Pakistan; i181613@nu.edu.pk (U.F.); muhammad.asim@nu.edu.pk (M.A.); 2 Department of Computer Science, Shaheed Zulfikar Ali Bhutto Institute of Science and Technology, Islamabad 44000, Pakistan; dr.noshina@szabist-isb.edu.pk; 3 Department of Computer Science, College of Computing and Informatics, University of Sharjah, Sharjah P.O. Box 27272, United Arab Emirates; tshamsa@sharjah.ac.ae; 4 College of Information Technology, United Arab Emirates University, Al Ain P.O. Box 17551, United Arab Emirates; 5 Department of Computer Science, Electrical and Space Engineering, Luleå University of Technology, 97187 Luleå, Sweden; 6 Faculty of Engineering, Al-Azhar University, Qena P.O. Box 83513, Egypt; 7 Centre for Security, Communications and Network Research, University of Plymouth, Plymouth PL4 8AA, UK

**Keywords:** Internet of Things, RPL, rank attack, Sybil attack, sinkhole attack, trust, mobile agent

## Abstract

Recently, the Internet of Things (IoT) has emerged as an important way to connect diverse physical devices to the internet. The IoT paves the way for a slew of new cutting-edge applications. Despite the prospective benefits and many security solutions offered in the literature, the security of IoT networks remains a critical concern, considering the massive amount of data generated and transmitted. The resource-constrained, mobile, and heterogeneous nature of the IoT makes it increasingly challenging to preserve security in routing protocols, such as the routing protocol for low-power and lossy networks (RPL). RPL does not offer good protection against routing attacks, such as rank, Sybil, and sinkhole attacks. Therefore, to augment the security of RPL, this article proposes the energy-efficient multi-mobile agent-based trust framework for RPL (MMTM-RPL). The goal of MMTM-RPL is to mitigate internal attacks in IoT-based wireless sensor networks using fog layer capabilities. MMTM-RPL mitigates rank, Sybil, and sinkhole attacks while minimizing energy and message overheads by 25–30% due to the use of mobile agents and dynamic itineraries. MMTM-RPL enhances the security of RPL and improves network lifetime (by 25–30% or more) and the detection rate (by 10% or more) compared to state-of-the-art approaches, namely, DCTM-RPL, RBAM-IoT, RPL-MRC, and DSH-RPL.

## 1. Introduction

The Internet of Things (IoT) is an advanced network for allowing interactions and communications among heterogeneous smart devices, such as sensors [1]. Data generated by these smart connected devices are assessed and collated, allowing users to make better choices and take appropriate action. With the proliferation of low-power sensing devices, many applications, including smart transportation, smart grids, smart homes, smart healthcare, and smart cities, are projected to benefit from IoT technology [2]. In addition, business and industrial applications are expected to account for as much as $11 trillion in the global economy by 2025, according to the McKinsey Global Institute [3]. Fog and edge computing, diverse network architectures, and various communication technologies, such as 5G and 6G, can provide enhanced availability and accessibility of information by using the IoT [4]. Low-power and lossy networks (LLNs) comprise a multitude of diverse and heterogeneous resource-scarce IoT devices. They help sense the surroundings, process the data sensed, and communicate wirelessly, offering a significant factor in PRESTO-emergent IoT applications.

Conventionally, LLNs use a standard routing protocol known as the routing protocol for LLNs (RPL). However, this protocol was not initially intended to safeguard systems against cyberattacks. Its security features are optional to deploy, since they can significantly reduce the effectiveness of resource-constrained devices. Hence, the constrained resources, openness, and distributed nature of such networks makes them vulnerable to many cyber- and routing attacks [5,6]. Normally, in a routing attack, such as rank, Sybil, blackhole, or sinkhole, a node exhibits abnormal behavior, such as selective forwarding or message tampering [7,8].

Sinkhole and Sybil attacks are the most damaging, since they can impede and prevent connections between network devices. In a sinkhole attack, a malicious node (i.e., an IoT device) will claim to be the perfect node for routing data to a target node. Thus, it presents false knowledge [9]. The node will illegitimately modify or drop traffic after it has obtained it. For instance, an attacker in RPL can exploit the ranking method by using misleading advertisements to advertise itself as a chosen routing node. The adversary promotes itself as being part of the best path and deceives other nodes into sending data via it. In this way, the adversary can initiate other network attacks and compromise network performance and lifetime. Similarly, a Sybil attack can reduce network lifetime by creating multiple identities and overwhelming the network with unwanted traffic. Therefore, this study assesses key internal attacks, particularly sinkhole and Sybil attacks, to create stable routing for IoT-based LLNs.

Furthermore, the latest security regulations (spam detection, data encryption, identity-authenticated key agreement, and digital signing) are inadequate for fixing IoT security problems [10,11]. These methods use considerable IoT resources and coercively impact device performance. Moreover, the cryptographic systems used to defend against external threats are ineffective when nodes are authenticated internally. Given these problems with RPL, trust-based security is highly recommended for mitigating internal attacks. Trust-based security can be pivotal in monitoring the forwarding behavior of nodes [12]. It can help in detecting malicious entities in a network to ensure secure and uninterrupted communications. However, most trust-based mechanisms consume power, communication bandwidth, and memory [10,11].

As well as the high overheads (i.e., power, message, and memory), most mechanisms do not consider the mobility of the nodes and networks. Furthermore, most IoT networks are dynamic by nature [1]. In a trust-based security framework, trust parameters are exchanged among nodes to compute the trust value, contributing to unwanted traffic and congestion. This approach increases energy consumption and message loss and deteriorates network lifetime and performance in terms of availability. Therefore, it is necessary to minimize network traffic when mitigating attacks.

First, there is a need to design a mechanism with minimal energy consumption at the node level to increase the network lifetime of LLNs. The energy levels of nodes must be conserved. For example, the Tmote Sky wireless sensor module can monitor temperature, light levels, and humidity. It has 48 kB of flash storage, 10 kB of RAM, and a 16-bit CPU with a battery of 30 kJ that is rarely recharged [13]. With RPL, such devices consume 0.0920 W per minute. Therefore, trust-related complex computations deplete these resources, and the sensor node will become exhausted.

Second, as well as control messages and sensed data, in a trust-based mechanism, trust-related data are also exchanged between nodes or sent to a central entity in a multi-hop fashion, causing unwanted traffic that leads to congestion.

In both cases, message overhead is generated, which must be minimized to maintain network performance. A high message overhead will deteriorate the performance of the network and may also increase delays and the packet loss ratio. In an LLN, nodes are always dynamic and mobile (i.e., they can join or leave the network), which is ignored in most state-of-the-art approaches. Most have a trust mechanism designed for a static environment. Therefore, it is necessary to design a mechanism that can cope with the dynamicity and mobility of the nodes and the environment.

Third, since the value of the trust (of a node) is highly dependent on the trust parameters, their integrity is extremely important. If the trust-related data (parameters) are compromised, the trust value will not depict the true situation. As a result, a malicious node may be identified as benign, leading to devastating consequences. Message floods, complex calculations, and memory overhead plague the majority of current experiments.

As a result, IoT networks, which have limited resources, can become ineffective. Moreover, because of the changing dynamics of the network, certain trust parameters cannot be adapted or scaled, which might have detrimental implications in battery-based RPL-based systems. Consequently, more node power is consumed, degrading the overall network lifetime. Therefore, it is necessary to develop trust parameters and strategies for low-power, resource-constrained, and lossy networks. Furthermore, ensuring the security of the data gathered is also crucial for the integrity of trust values.

### 1.1. Research Questions

To improve RPL security against internal attacks, this work poses the following research questions (RQs) based on the research gaps found and discussed in Section 2.1:

RQ1. How can security in RPL be improved to mitigate internal attacks while maintaining the mobility of the network?
RQ2. How can we ensure that data are gathered securely to protect RPL-based networks against internal attacks?
RQ3. In support of RQ1, can we minimize network congestion (overhead due to the number of messages exchanged) to improve network lifetime and minimize energy, memory, and computational overheads at the device level in RPL-based infrastructures?

### 1.2. Our Contributions

This study proposes the multi-mobile agent-based trust framework for RPL (MMTM-RPL) to mitigate internal attacks (i.e., sinkhole, Sybil, and rank attacks) and support the use of RPL in LLNs. It reduces the message overhead using mobile agents (MAs) and minimizes energy utilization using a fog layer, which performs all the computing and analysis. The major contributions of this paper are as follows:

- A novel security framework for mitigating Sybil, rank, and sinkhole attacks in RPL-enabled LLNs is presented.
- Authenticated multiple MAs and dynamic itineraries minimize the message overhead and improve efficiency.
- A framework with a fog layer is presented in detail. It minimizes energy, computation, and memory overheads at the node level, which increases network lifetime.

### 1.3. Organization of This Paper

The rest of the paper is organized as follows. Related work is discussed in Section 2. The proposed framework is described in Section 3. Section 4 illustrates the evaluation and results of the proposed framework, and a discussion of the results is given in Section 5. The conclusions and future work are presented in Section 6.

## 2. Related Work

Cyberattacks against the IoT occur at many IoT stack levels, making the IoT a target for new assaults. In response, researchers have concentrated their efforts on creating different mechanisms to increase the security, productivity, and effectiveness of the IoT. To protect communications in the IoT network layer, ref. [14] suggested a trust-based routing protocol for identifying and segregating routing attacks in IoT networks. Similarly, an intrusion detection system based on a deep neural network was proposed in [15]. It identifies internal assaults on medical IoT networks. A multi-agent model was proposed using deep learning in [16]. Many researchers have extensively studied the vulnerabilities of RPL, including various internal attacks, for instance, Sybil, rank, sinkhole, version number, blackhole, neighbor, DIS, DAO, and selective-forwarding attacks [14,17,18,19]. (DIS and DAO are explained in Section 3.1.2). The authors in [20] designed a cryptography-based security measure to mitigate rank and version-number assaults using a hash chain, and malicious nodes were removed from the network using this rank chain.

In the approach suggested by [21], all network nodes are registered with a security organization. The frequent demise of nodes was addressed by introducing a mobile sink. To mitigate rank attacks, before any grid member node can send data to the grid head, it must be authenticated. A different mechanism was proposed in [22] based on layering to defend RPL against a rank assault. The suggested approach has three stages: (1) Nodes are classified into layers. (2) Route trust values are computed. (3) Rank attacks are identified and isolated. A sinkhole attack against RPL was detected using ranking and rating techniques in [23]. They calculated distances to determine the rank and rate of each node. Additionally, the average packet transmission route request (APT-RREQ) value identifies nodes in the IoT network behaving oddly.

Tandon and Srivastava [24] proposed a collective trust mechanism for secure routing in IoT-based infrastructures. Their mechanism protects against rank and Sybil attacks. Although the proposed mechanism minimized energy consumption and increased detection accuracy and throughput, it incurred a high computational and message overhead. In [18], a time-based trust-aware routing protocol called SecTrust-RPL was proposed to mitigate rank and Sybil attacks. The proposed mechanism improved network performance and isolated internal attacks successfully. However, it ignored the energy overhead. Zaminkar et al. [23] proposed a novel protocol, SoS-RPL, for mitigating sinkhole attacks. It has two phases: (1) nodes are rated and ranked based on distance measurements and (2) misbehaving nodes and sources are identified based on APT-RREQ. However, the proposed mechanism did not reduce the message overhead and ignored the mobility of the network.

Zaminkar et al. [25] proposed SybM to mitigate mobile Sybil attacks and safeguard RPL. They allowed for node mobility and handled flooding fake control messages. However, the proposed mechanism has a lower accuracy and detection rate than other state-of-the-art systems. Iqbal et al. [26] proposed a trust-based mechanism for detecting rank and sinkhole attacks in multi-sink networks. It is an energy-efficient mechanism. However, they did not consider the message and computational overhead. Similarly, in [27], a robust hybrid and encryption-based mechanism was proposed to secure nodes in RPL-based infrastructures. The security mechanism has four phases. However, the computational and energy overhead was high. Pu et al. [5] presented a mechanism using a Gini index to mitigate Sybil attacks in RPL-based LLNs. However, the proposed mechanism did not reduce the message overhead.

Hashemi and Aliee [28] proposed a dynamic trust-based mechanism called DCTM-IoT and an RPL-based Sybil attack mitigation system called DCTM-RPL. However, these proposed mechanisms have high message and energy overheads. A method for detecting rank attacks [29] targeted the latter process in RPL topologies. A trust threshold was established based on the rankings of surrounding nodes. Compared to RPL not under and under attack, the simulation results demonstrated that the suggested method was more efficient than the state-of-the-art approach.

In [25], homomorphic encryption is proposed for data transmitted between the root and the objects to ensure that hostile nodes cannot intercept the data. The private key is used to recover the primary message, and the public and private keys are used for messages to nodes. Several IoT scenarios use the current version of the proposed DSH-RPL architecture. A method proposed in [30] mitigated the same problem by lowering power usage and the control and data-packet overheads. This scalable framework was designed to safeguard RPL-LLN. The authors proposed combining their technique with additional detection methods, such as threshold-based detection. It can prevent multicast DIS attacks well before the attackers are otherwise spotted and thrown out of the network.

### 2.1. Critical Analysis and Research Gaps

Table 1 is a summary of different state-of-the-art approaches. It shows whether the mobility of the network has been catered for. A 🗸 represents the corresponding feature is present or high (e.g., a 🗸 for message overhead means it is high and a 🗸 for mobility means it is addressed), and a × represents a low or the absence of a feature (e.g., a × for message overhead means it is low and a × for mobility means it is not addressed). Similarly, it compares energy, message, and computational overheads. It also compares network performance and network lifetime with those achieved by the proposed approach. Only [27,28] considered node and environment mobility.

References [25,27,28] proposed an energy-efficient trust mechanism in which most of the calculations are done by the root node. However,  the authors in [25] used homomorphic encryption, which increased the computational overhead on nodes during decryption. The message overhead is also higher because no effective transmission method was used from the root to a node or vice versa. Similarly, the authors in [27,28] calculated the trust at the node level, but there was no mechanism for exchanging messages, resulting in unwanted message and computational overheads. These state-of-the-art approaches do not minimize the message overhead while detecting and isolating internal attacks and performing trust-related calculations by device layers. The studies in [25,27,28] ensured that the network lifetime was long by improving energy efficiency.

This analysis shows that most state-of-the-art approaches aim to reduce energy consumption and increase network lifetime. However, they ignore traffic congestion (i.e., number of messages exchanged) and computational overheads, which are the main causes of low network performance. Mobility is also a crucial feature of LLN-based IoT networks [31], but only a few methods cater for it.

For a network, its lifetime is considered to end at the death of the first node. To increase network lifetime, energy must be conserved by nodes. For LLNs, therefore, there is a need to design a mechanism that minimizes energy consumption at the node level.

Moreover, as well as messages containing sensed data, in a trust-based mechanism, trust-related data are also exchanged between nodes or sent to a central entity in a multi-hop fashion, causing unwanted traffic and leading to congestion. This increases the message overhead, which must be reduced to improve network performance and reduce delays and the packet loss ratio.

To overcome these issues and limitations, this research introduces MMTM-RPL, which aims to improve the security of RPL-based LLNs. It maximizes network lifetime by conserving energy at the node level and improves network performance by minimizing network congestion at both node and channel levels. All the complex trust-related computations and storage are done at the fog or control layer to conserve resources.

To reduce network congestion caused by exchanging trust parameters, MMTM-RPL uses MAs, which were recently employed in wireless sensor networks to conserve energy and improve data collection [32]. The basic purpose of data aggregation is to capture, gather, and analyze data efficiently. Finding the best itinerary for the MAs is a key stage in data collection [33]. However, as the network scale grows, using a single MA itinerary suffers from two drawbacks: delay and its huge size. To address these issues for a wireless sensor network, MMTM-RPL has an efficient data aggregation technique that leverages numerous MAs to aggregate data and delivers the data to the sink depending on the planned itinerary. It also ensures that data gathering is secure by using integral and valid trust values to diminish the chances of the misapprehension of a malicious node. The underlying network is divided into different clusters with dynamic itineraries. Each MA visits its corresponding cluster to fetch the trust parameters.

## 3. Proposed Framework

This section details the proposed layer-wise architecture and the steps and flow involved in detecting and isolating internal attacks.

### 3.1. MMTM-RPL Layers

There are two layers in the proposed framework: a device layer and a control layer. The details of each layer are discussed below.

#### 3.1.1. Device Layer

**Figure 1:**
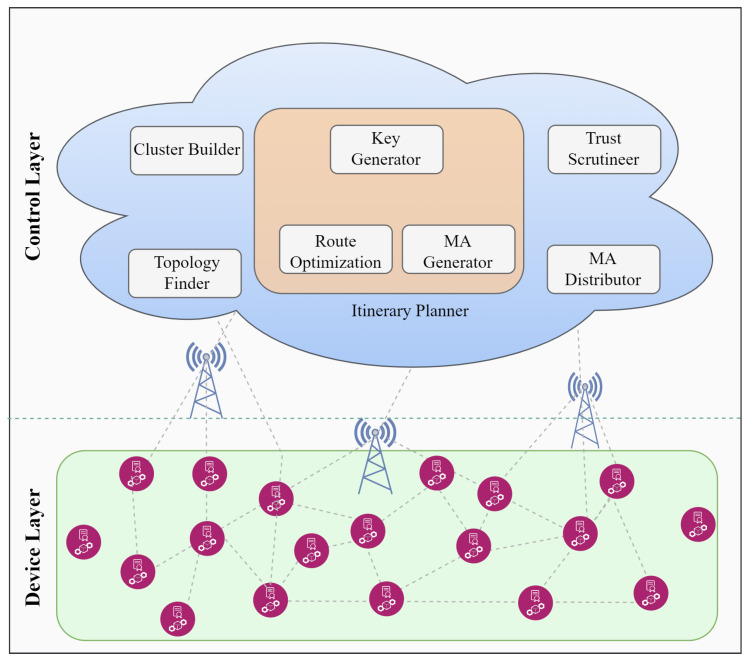
Proposed device and control layers.

This layer consists of LLN-based IoT devices (e.g., sensors), which are well known for their constrained nature (Figure 1). There is a dire need to conserve their limited resources to enhance network lifetime and performance. Since these devices are highly resource-constrained and use RPL (which is not secure against internal attacks), ensuring their security is challenging. Therefore, MMTM-RPL aims to minimize the processing, storage, and energy drain for such devices while enhancing the security of RPL. To mitigate internal attacks, such as rank, Sybil, and sinkhole (in our scenario), these devices provide trust-related data to the upper layer (via an MA), which detects and isolates malicious nodes.

#### 3.1.2. Control Layer

Because of the low resources of the devices, the control layer (i.e., the fog layer) performs most of the operations and deals with internal attacks (Sybil, rank, and sinkhole attacks in our case). It performs various operations and has several modules (Figure 1), which are discussed in detail below.

##### Topology Finder Module

**Figure 2:**
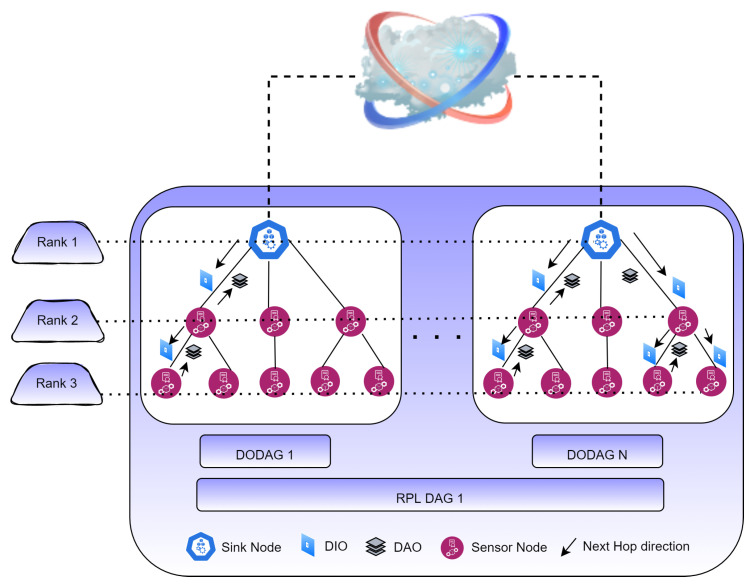
Topology discovery in RPL.

The topology finder module finds the topology and nodes of the underlying device layer (Figure 2). It creates destination-oriented directed acyclic graphs (DODAGs) based on an objective function (OF), for instance, delay, energy used, or hop count [34]. Generally, each RPL instance has an optimization goal that depends on the application goal. The application goal is represented by the OF, such as Zero-OF or MRHOF [35]. The RPL topology is organized into one or more DODAGs, each rooted at a single point, the DODAG root (or sink node). It uses distance vector routing.

When the process begins, a tree-like structure known as a directed acyclic graph (DAG) is formed, and a parent node is selected by each node [18]. The corresponding parent node is the gateway for its child nodes. Information about the DAG is maintained by each DODAG, which each node receives from its parent. Control messages, such as DODAG information solicitation (DIS) messages, DIO acknowledgments, DODAG information objects (DIOs), and destination advertisement objects (DAOs), are transmitted [36]. All these message types are used to build the topology step by step. The parent broadcasts DIO messages to inform nodes about it. Following the acquisition of a parent node, all nodes rate themselves using the OF. DAO messages are sent from all nodes that have received DIOs. By transmitting DIS control messages, nodes not yet in the DODAG topology can become connected [37,38].

When an RPL instance sets up routes in the whole network, the OF is calculated. RPL instances join each DODAG many times. RPL calculates the rank of each node and how many sink nodes there are. The rank of a node indicates its distance from its parent node. Child nodes must have a higher rank than their parent and are announced across the network to prevent loops. To link an LLN to the internet or any other network, a node often uses an IPv6 Border Router/gateway. Nodes can be controlled by one or more RPL instances.

The topology finder module uses the MRHOF OF of RPL and a trickle timer algorithm. It recovers the topology over time for a wireless sensor network or the IoT [39]. Other than periodic topology updates, every time the location of a node changes (i.e., it leaves a cluster and enters the perimeter or range of another), the topology finder module tells the cluster builder to take the appropriate steps [40].

##### Cluster Builder Module

In this module, the underlying network found by the topology finder module is divided into independent clusters. In RPL, the absence of load balancing results in an uneven allocation of network traffic and reduces network efficiency. The proposed framework utilizes cluster-tree RPL, which was used in [41] for selecting the cluster head and setting routes using the Euclidean distance. It also utilizes fuzzy *C*-means clustering (FCM) to evaluate the geographical location and energy level of each node. Clustering techniques that employ FCM are among the most frequently used soft clustering algorithms. Other types of clustering, such as *k*-means, can be inferior to FCM. For example, in the *k*-means algorithm, there is a probability that a data point can belong to many clusters. For overlapping data sets, FCM clustering yields superior results [40].

FCM determines an anticipated number when generating a cluster. It provides the centroid value of such clusters by utilizing the node membership value [42]. The cluster builder module calculates clusters periodically or on discovering changes to the network topology. The value of the membership node D(pq) depends on the Euclidean distances from all the cluster centroids:

(1) $$D\left(pq\right)={\left[\sum_{k=1}^{M}\frac{d_{pq}^{2}}{d_{pk}^{2}}\right]}^{-1},\;p=1,2,3\ldots N,\;q=1,2,3\ldots M$$

where *p* and *q* are two nodes and *d* is the Euclidean distance between them. Here, *N* and *M* are the number of nodes in the sensor field and the number of nodes in the available clusters, respectively. The Euclidean distances are measured by each node in the network [36]. The distance ED(pq) is computed for two nodes:

(2) $$ED\left(pq\right)=\sqrt{{\left(p_{m}-p_{l}\right)}^{2}+{\left(q_{m}-q_{l}\right)}^{2}}$$

where *l* and *m* are two points with coordinates $\left(p_{l},q_{l}\right)$ and $\left(p_{m},q_{m}\right)$, respectively. The distance matrix:

(3) $$Distance=\left[\begin{array}{cccc} 0 & Distance\left(1,2\right) & \cdots & Distance\left(1,n\right)\\ \vdots & 0 & \cdots & \vdots \\ Distance\left(n,1\right) & Distance\left(n,2\right) & \cdots & 0 \end{array}\right]$$

The centroid is computed as follows:

(4) $$\mathrm{Centroid}\left(\left(p_{l}-q_{l}\right),\left(p_{m}-q_{m}\right)\right)=\left(\frac{p_{l}+p_{m}}{2},\frac{q_{l}+q_{m}}{2}\right)$$

At the start, the centroid point has the coordinates of the first node. The distance ED(pq) between that node and the other node is calculated by CT-RPL [41], and the centroid of the two nodes is also found. If the centroid falls within distance ED(pq), the node is added to the cluster. Otherwise, it is added to a distant cluster.

The number of clusters is calculated as follows:

(5) $$N_{\left(\mathrm{clusters}\right)}=\left\{\begin{array}{cc} \mathrm{Centroid}(p_{l},q_{m})\pm \mathrm{Distance}, & \mathrm{for}\;\mathrm{the}\;\mathrm{same}\;\mathrm{cluster},\\ \mathrm{Distant}\;\mathrm{cluster}, & \mathrm{otherwise} \end{array}\right.$$

The total number of clusters:

(6) $$N=\sum_{i=1}^{n}=\mathrm{Count}\left(\mathrm{Centroid}\left(p_{l},q_{l}\right)\right)$$

where *n* is the number of nodes in a cluster. A node with the maximum value is added to the respective cluster. The FCM algorithm provides authentic clustering via OF minimization:

(7) $$obj\left(f\right)=\sum_{k=1}^{N}\sum_{j=1}^{n}D_{\left(kj\right)}^{fm}d_{\left(kj\right)}^{2}$$

The fuzziness parameter is fm, which is a very important parameter in clustering, and its value is always greater than 1. In our case, its value is ${}_{4}^{1}2$, which is also used in normal circumstances.

In short, the initial step after deploying RPL-Topology is to create clusters in that topology. Then, the FCM is used to cluster the nodes, as illustrated in Algorithm 1. It uses seven steps to complete the tasks:
**Algorithm 1** Fuzzy *C*-means clustering.1:parameters: $p,n,N,fm,p_{l},p_{m},D^{fm},d$2:initialize: $p,n,N,fm,p_{l},p_{m},D^{fm},d$3:randomly initialize the membership matrix:
$$\;\;\;\;\;\;\;\;\;\;\;\;\;\;\;\;\;Distance=\left[\begin{array}{cccc} 0 & Distance\left(1,2\right) & \cdots & Distance\left(1,n\right)\\ \vdots & 0 & \cdots & \vdots \\ Distance\left(n,1\right) & Distance\left(n,2\right) & \cdots & 0 \end{array}\right]$$4:calculate the centroid:
$\;\;\;\;\;\;\;\;\;\;\;\;\;\;\;\;\;\;\;\;\;\;\;\;\mathrm{Centroid}\left(\left(p_{l}-q_{l}\right),\left(p_{m}-q_{m}\right)\right)=\left(\frac{p_{l}+q_{m}}{2},\frac{q_{l}+q_{m}}{2}\right)$5:calculate the Euclidean distance:
$\;\;\;\;\;\;\;\;\;\;\;\;\;\;\;\;\;\;\;\;\;\;\;\;EU\left(pq\right)=\sqrt{{\left(p_{m}-p_{l}\right)}^{2}+{\left(q_{m}-q_{l}\right)}^{2}}$6:update the new membership matrix:
$\;\;\;\;\;\;\;\;\;\;\;\;\;\;\;\;\;\;\;\;\;\;\;\;obj\left(f\right)=\sum_{k=1}^{N}\sum_{j=1}^{n}D_{\left(kj\right)}^{fm}\cdot d_{\left(kj\right)}^{2}$7:**repeat**8:    step 49:**until** centroids no longer change10:**while** the topology has changed **do**11:    steps 1 to 612:**end while**

1. The variables used are set up.
2. The membership matrix of each node is initialized. This matrix is used to determine the routes to its neighbors.
3. The centroid is found once the membership matrix is complete.
4. The centroid is based on the predefined region or area for a cluster.
5. The Euclidean distance is calculated as the distance between a node and its centroid node.
6. The membership matrix is updated using the computed distance.
7. The gateway is given a unique secure key (hashed using Message Digest 5 (MD5)) to authenticate MAs.

These steps in the calculation of the centroid are repeated until the centroid does not change or a node joins or leaves a cluster or the network.

##### Itinerary Planner Module

The itinerary planner then generates the same number of MAs as there are clusters and assigns an individual itinerary to each MA. It also allocates a security key to each MA, which helps the gateway to verify the MA by using hashed key values. If the keys match, the MA is allowed to collect data (i.e., trust parameters) from the respective cluster for trust evaluation. Otherwise, it is not allowed, and a new MA is initiated dynamically. The itinerary planner module has different sub-modules for these activities, as discussed below:

1. Route Optimization: A routing protocol is needed to evaluate the intended network to minimize power consumption to maximize the network lifespan. There are many routing protocols. One is the minimum spanning tree (MST) algorithm, which optimizes power use by reducing communications overall. Therefore, the proposed framework uses an MST. This is a tree where the nodes in each vertex pair are linked by only one path. There is a single tree spanning the nodes with the lowest total edge length. The tree with the lowest span is a weighted tree subgraph, which is a subgraph without loops [43]. It is a non-expanded and linked graph. A tree is often an extensive unit distance graph (UDG) that has edges that link all nodes inside the communications range, $R_{c}$:
  (8) $$G_{\left(UDG\right)}=\left(V,\left\{\left(l,m\right)|\left(l,m\right)\in E\;\mathrm{and}\;0<\delta \left(pos\left(l\right),pos\left(m\right)\right)<R_{c}\right\}\right)$$
  where
  $$\delta \left(pos\left(l\right),pos\left(m\right)\right)=\sqrt{\Delta X_{l,m}^{2}+\Delta Y_{l,m}^{2}}$$
  and pos represents the location of the sensor nodes in $R_{c}$. The edges linking all *n* sensor nodes to the n−1 edges in the MST have the lowest total weight. The weights are equivalent to the edge lengths. In this instance, the total MST edge length must be minimal.
  Algorithm 2 provides the steps for route optimization. Once a cluster is full, the algorithm finds the shortest path between each pair of nodes within the cluster. To do so, it calculates routes for each cluster using the MST. It starts by calculating the hop count from the centroid. Initially, hop_count is set to 0. If hop_count is 1, it is incremented by 1; otherwise, it is incremented by 2. Then, if the hop count of a node is less than 2, it is chosen for routing; otherwise, a node with a lower hop count is chosen.
  Algorithm 3 shows the steps used in dynamic itinerary management and for the security checks for MAs. First, an MA is generated for each cluster. Then, the itinerary planner generates and manages routes established by the MST algorithm and assigns security keys to each MA. An MA is first validated against its identity held by the gateway before it can enter its cluster. If an MA is valid, the gateway lets it visit the cluster and acquire the (trust-related) data from each node listed in its itinerary. Otherwise, it is discarded, and a new MA is then initiated with a new security key.
2. Key Generator: This module is responsible for securely gathering the trust-related data. When an MA is initiated, a unique key is assigned to it, and this key is sent to the gateway of its cluster. The key is hashed with MD5 by the control layer. Each time an MA visits the device layer, the hashes are crossed-checked. If authenticated, it can enter the respective cluster to fetch the trusted parameters.
  Using MD5 poses no significant risks since it is a sophisticated and efficient file hash and is a faster and more secure cryptographic hash algorithm than some other functions. For instance, the performance of a hash function can be assessed by its complexity and execution time. MD5 and SHA-256 both have the same complexity of O(N), but MD5 is faster than SHA-256. Although MD5 is prone to collisions, some researchers have proposed different ways to overcome collisions [44,45,46].
  The primary advantage of MD5 is that it takes inputs of any size and outputs a 128-bit hash or message digest [47]. It processes each 512-bit block of data separately, including the message digest from the preceding stage. Each 512-bit string is split into 16 words, each of which has 32 bits. Message digests are created with consecutive hexadecimal numbers in the first phase of initialization [48]. MD5 uses message padding to adjust the length.
3. MA Generator: This module generates multiple MAs based on the number of clusters made by the cluster builder module. It is also responsible for initiating all the MAs when required. The MAs begin topology discovery whenever an MA security hash fails or a change in topology is discovered. After the MAs are initiated, they are sent to the MA distributor module.

**Algorithm 2** Route optimization with the minimum spanning tree algorithm.1:initialize variables:2:hop_count=03:$x_{a}$←$node_{a}$4:$x_{b}$←$node_{b}$5:centroid node6:C ← cluster7:**for all** clusters (C) **do**8:    calculate hop_count from centroid9:    **if** distance between ($x_{a}$ and centroid) == 1 **then**10:        hop_count=hop_count+111:    **else**12:        hop_count=hop_count+213:    **end if**14:    **if** hop_count<2 **then**15:        select node for routing16:    **else**17:        drop node18:    **end if**19:**end for**20:**repeat**21:    step 722:**until** the centroid does not change23:**while** the topology has changed **do**24:    steps 1 to 725:**end while**

**Algorithm 3** Dynamic itinerary planner algorithm.
1:initialize variables:2:MA = 03:itinerary4:MA = Number of populated clusters5:**for all** clusters **do**6:    initialize security parameter7:
**end for**
8:key ← security variable9:
**if**
10:    key == 0 **then**11:    hashed key ← MD5(key value)12:    MA ← hashed key13:    Gateway (G) ← hashed key14:
**end if**
15:**for all** clusters (C) **do**16:    **function**17:        call ← Routing()18:        Routing() ← MST algorithm19:        itinerary ← every cluster20:        assign every cluster itinerary to MAs21:        MA ← itinerary22:    **end function**23:
**end for**
24:**for all** clusters (C) **do**25:    MA → visit a cluster26:    **if** MA hashed key == Gateway hashed key **then**27:        authenticate MA28:        collect data29:    **else**30:        invalid key31:    **end if**32:
**end for**
33:**for all** clusters **do**34:    steps 15 and 2435:
**end for**
36:**while** the topology has changed **do**37:    steps 1 to 2438:
**end while**



##### MA Distributor Module

This module receives multiple MAs from the MA generator module and directs them to the device layer for gathering trust-related data (i.e., trust parameters). Once an MA successfully fetches the data, it heads back to the control layer and reports back to this module, which forwards these details to the trust scrutineer module for trust assessment.

##### Trust Scrutineer Module

Subjective logic (SL) [49] is a subset of logic that enables the manipulation of subjective beliefs or opinions. The degree of uncertainty of an opinion indicates the probability of it being true. SL specifies a collection of procedures that can be performed on opinions. It is an extension of the conventional belief function model. Additionally, this logic is distinct from fuzzy logic, which accurately uses imprecise propositions. Because SL is based on regression analysis, it is well suited for fog computing. Conventionally, SL-based trust is built on individual trust (i.e., the trust value of each node is calculated independently based on its behavior). Each node is responsible for computing the trust value of its neighboring nodes. Every node keeps an eye on the behavior of its neighboring nodes and shares trust-related data (i.e., trust parameters). If the calculated trust value for a node is less or greater than some threshold (depending upon the application design), it is identified as an attacker and isolated from the network. However, among other drawbacks, each node has to compute, analyze, and store all the trust values, which consumes quite a considerable amount of resources, including memory, computation, storage, and energy [50].

Therefore, in MMTM-RPL, trust is computed and analyzed by the control layer to minimize resource utilization. It uses the same SL features as a preventative method. However, not all evidence of trust may be accessible, making a judgment ambiguous. SL is founded on belief theory. At a given time, it trusts just one of the potential system states. The belief model indicates whether node $N_{i}$ is genuine. It establishes the trust value for each node depending on how the node is perceived. This perspective comprises values for disbelief $d_{s}$, belief $b_{l}$, and unknown $u_{n}$:

(9) $$b_{l}+d_{s}+u_{n}=1$$

Here, $b_{l}$ indicates that the object is in the trusted state, $d_{s}$ indicates that the object is not trustworthy, and $u_{n}$ indicates that trust in this node is unspecified or unsure.

A prior trust indication for each node that lacks explicit proof is stored using Equation (9). Additionally, the value is contingent upon whether a node is pre-trusted and recognized during the early phases of the system. Prior trust is critical for a node that has just entered the network [51]. It is dependent upon this criterion:

(10) $$\alpha =\left\{\begin{array}{cc} 1, & \mathrm{if}\;\mathrm{the}\;\mathrm{node}\;\mathrm{is}\;\mathrm{trusted}\\ 0.5, & \mathrm{if}\;\mathrm{the}\;\mathrm{node}\;\mathrm{is}\;\mathrm{distrusted} \end{array}\right.$$

where the base rate α represents the uncertainty in the level of belief or disbelief.

The level of trust in a node $N_{i}$ is expressed as follows:

(11) $$w_{i}=\left(b_{l}^{i},d_{s}^{i},u_{n}^{i}\right)$$

For positive (i.e., fair) and negative (i.e., a breach) experiences, the trust values can be evaluated as follows:

(12) $$\begin{array}{cc} b_{l} & =\frac{p_{s}}{p_{s}+n_{g}+1} \end{array}$$

(13) $$\begin{array}{cc} d_{s} & =\frac{n}{p_{s}+n_{g}+1} \end{array}$$

where $p_{s}$ is the number of positive or good experiences and $n_{g}$ is the number of negative or bad experiences.

SL aims to establish the trustworthiness of a node before any data are shared with it. Factors $b_{l}$, $d_{s}$, and $u_{n}$ all impact whether or not our self-experience is accurate. It is a recommendation or suggestion, and the weight of the overall recommendation must be computed to confirm whether the advice is correct. It is determined using either the discounting or consensus method. In the discounting method, a discounting operator ⊕ is used. If node *X* wishes to calculate the trust of node *Y*, then it may use suggestions from the other network nodes.

Consider that *X* has an opinion about *Y* and that *Z* heeds this opinion. The overall trust for *Y* is a combination of the trust that *X* has in *Y* and the trust that *Z* has in *X*. The trust that *Z* has in *X* can be calculated as:

(14) $$\begin{array}{c} w_{X}^{Z}=\left(b{{}_{l}{}_{X}}^{Z},d{{}_{s}{}_{X}}^{Z},u{{}_{n}{}_{X}}^{Z}\right) \end{array}$$

The trust that *X* has in *Y* can be calculated using

(15) $$\begin{array}{c} w_{Y}^{X}=\left(b{{}_{l}{}_{Y}}^{X},d{{}_{s}{}_{Y}}^{X},u{{}_{n}{}_{Y}}^{X}\right) \end{array}$$

The trust that *Z* has in *Y* is then:

(16) $$\begin{array}{c} w_{Y}^{Z}=w_{X}^{Z}⊕w_{Y}^{X}=(\left(b{{}_{l}{}_{X}}^{Z}b{{}_{l}{}_{Y}}^{X}\right),\left(b{{}_{l}{}_{X}}^{Z}d{{}_{s}{}_{Y}}^{X}\right),\left(d_{s}{{}_{X}}^{Z}+u{{}_{n}{}_{X}}^{Z}+b{{}_{l}{}_{X}}^{Z}u{{}_{n}{}_{Y}}^{X}\right)) \end{array}$$

In the proposed model, the opinion about prop (i.e., the propositions of nodes as trustworthy or untrustworthy or uncertain trust value) from op (i.e., an opinion set) using *M* (i.e., a binomial variable as true or false) is then:

(17) $$M_{prop}^{op}=\left\{b_{prop},d_{prop},UM_{prop},autoM_{prop}\right\}$$

where $b_{prop}$, $d_{prop}$, $UM_{prop}$, and $autoM_{prop}$ represent belief, disbelief, uncertainty, and the rate of atomicity, respectively. In particular, SL is used to compute the confidence between different network entities, where confident measures can be expressed as beliefs. It may also be used analogously to compute the reputation of nodes in sensor-based networks. Moreover, it can also be utilized for other applications.

Algorithm 4 illustrates the proposed trust model for detecting and isolating malicious nodes. It uses the subjective logic framework (SLF) for assessing the trust value of a node. The parameters used in the trust computation and evaluation are initialized. First, the values of α and β are set. These represent the maximum packet drop ratio (PDR) and the maximum MAC address per unit time, respectively. The model uses Rank (i.e., the RPL-DODAG variable), PDR, and ρ (i.e., the rate of change of MAC address per unit time) as trust parameters. The MAC address is divided by the time slot *d* (i.e., the time slot to check the *MAC* address) to obtain ρ (i.e., the trustworthiness of a node). All the other parameter values are set to 0 initially. Positive (*p*), negative (*n*), and uncertainty (*u*) values are calculated using these parameters in SLF.

For each node, if PDR and ρ are higher than the relevant threshold (i.e., α or β), the packet has been lost. This illustrates that the *MAC* value is always changing. As a result, the negative count, which was initially set to 0, is incremented; otherwise, the positive count is incremented. Next, the disbelief, uncertainty, and belief values are computed based on these negative and positive counts. If the belief value is greater than the threshold (i.e., 0.5), the node is trustworthy. If not and if the disbelief value exceeds a threshold (also 0.5), the node is malevolent (i.e., a sinkhole, Sybil, or rank node in our case). Therefore, if disbelief in a node is high, it is removed from the topology; otherwise, it is used for routing.

### 3.2. The Workflow at a Glance

**Figure 3:**
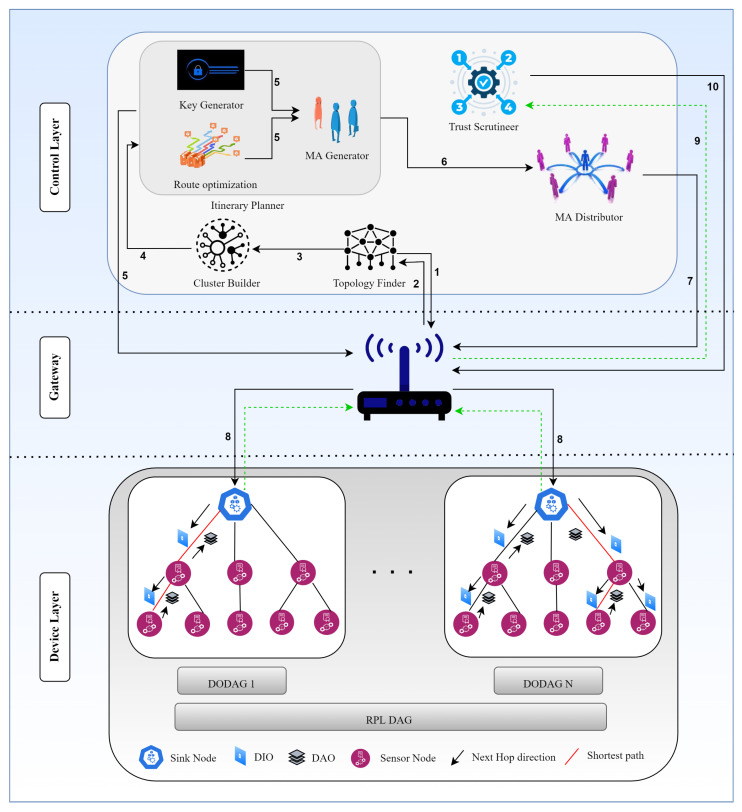
Workflow of the proposed MMTM-RPL framework. The numbers in the figure represent the steps taken for malicious node detection and isolation.

The architecture and the steps involved in MMTM-RPL are illustrated in Figure 3 and Algorithm 5, and the flow is listed below:

1. The topology finder module sends a request to the gateway to deploy the RPL topology.
2. The gateway deploys the topology, and details are sent back to the topology finder module, which forwards these details to the cluster builder module. The topology finder looks for changes to the topology (i.e., nodes joining and leaving). It updates the cluster builder module as soon as it identifies a change.
3. The cluster builder module creates logical clusters based on Euclidean distance. If the distance for any node is lower than a threshold, it is dropped from the cluster. This task is repeated every time a node joins or leaves the network dynamically.
4. Once the clusters are created, their details are forwarded to the itinerary planner.
5. The route optimizer module, a sub-module of the itinerary planner, looks for optimized paths in the given RPL based on the logical clusters. Once these are found, the itinerary planner module sets all the details of them for each cluster. Multiple MAs are created by the itinerary planner module for each cluster, and a unique MD5 hash key for a cluster is assigned to each MA by the security provider module. The same unique key is shared with the gateway for (later) MA verification.
6. The MAs are forwarded to the MA distributor module.
7. The MA distributor module sends the MAs to the gateway, which authenticates each MA using the unique key set earlier by the security provider module. If an MA fails authentication, a new MA is initiated dynamically for the itinerary.
8. Once an MA is authenticated, it is allowed by the gateway to visit its specified cluster to fetch trust-related data.
9. After an MA has collected data, the gateway forwards it to the trust scrutineer module.
10. The trust scrutineer module calculates the trust for each node. If a node is deemed to be malicious, it is dropped from the network.

**Algorithm 4** Malicious node detection and isolation.1:parameters: α, β, *p*, *n*, *k*, *d*2:initialize variables:3:α = threshold for max. PDR4:β = threshold for max. changing *MAC* address5:$p_{s}=0$6:$n_{g}=0$7:k=28:d= time slot9:trust parameters: RANK, PDR, ρ10:calculate trust parameters:11:RANK = RPL-DODAG                                                        ▹ initialize rank of each node12:$PDR=\left(\frac{\mathrm{dropped}\;\mathrm{packets}}{\mathrm{total}\;\mathrm{no}.\;\mathrm{of}\;\mathrm{packets}}\right)$13:$\rho =\frac{MAC}{d}$14:**for all** nodes **do**15:    evaluate sinkhole:16:    **if** PDR>α **then**17:        $n_{g}=n_{g}+1$18:    **else**19:        $p_{s}=p_{s}+1$20:    **end if**21:    evaluate Sybil:22:    **if** ρ>β **then**23:        $n_{g}=n_{g}+1$24:    **else**25:        $p_{s}=p_{s}+1$26:    **end if**27:    evaluate rank:28:    **if** $Rank_{xi}==Rank_{xj}$ **then**29:        $n_{g}=n_{g}+1$30:    **else**31:        $p_{s}=p_{s}+1$32:    **end if**33:**end for**34:calculate $b_{l}$, $d_{s}$, and $u_{n}$:35:$b_{l}=\left(\frac{p_{s}}{\left(p_{s}+n_{g}+k\right)}\right)$36:$d_{s}=\left(\frac{n}{\left(p_{s}+n_{g}+k\right)}\right)$37:$u_{n}=\left(\frac{k}{\left(p_{s}+n_{g}+k\right)}\right)$38:node rating or malicious node detection:39:**if**$\;b_{l}>0.5\;$**then**40:    node → legitimate node41:**else if**$\;d_{s}>0.5\;$**then**42:    node → sinkhole/Sybil/rank/malicious43:**else**44:    node ← uncertain45:**end if**46:node mitigation:47:**if** node == attacker **then**48:    remove from topology49:**else**50:    select for routing51:**end if**

**Algorithm 5** RPL-based trust algorithm.
1:initialize variables:2:initialize all FCM variables3:initialize all MST parameters4:initialize all trust parameters5:
**function**
6:    Call ← RPL topology()7:
**end function**
8:
**function**
9:    Call ← Clustering()10:    Clustering() ← FCM algorithm11:
**end function**
12:
**function**
13:    Itinerary() ← dynamic itinerary planner algorithm14:    Call ← Routing()15:    Routing() ← MST algorithm16:    Call ← mobileAgent()17:    mobileAgent() ← MA generator18:    Call ← Security()19:    Security() ← Security key generator and assignment20:
**end function**
21:
**function**
22:    Call ← Trust()23:    Trust() ← trust SLF algorithm24:    Trust propagation of malicious node isolation25:
**end function**



## 4. Experimental Works and Results

The experimental setup and findings in terms of network lifetime, average residual energy, message overhead, and end-to-end delay are discussed in this section.

### 4.1. Experimental Setup

The proposed model was simulated using the Cooja network simulator. A lightweight SLF was utilized as the trust model in the fog layer. A modified Cooja platform based on Contiki OS was used for the implementation [12]. This OS was installed on a laptop with an Intel^®^ Core^TM^ i5-3317U CPU running at 1.70 GHz with 8 GB RAM. The suggested model was tested with various scenarios, including small and large networks. With the same initial energy of 100 J, we set up a network with 30 to 120 sensor nodes. Data and control messages were randomly sent between nodes. The number of malicious nodes ranged from 3 to 12 with a ratio of 1:100. Table 2 summarizes the parameters used in the simulation and experimentation along with the set values.

#### 4.1.1. Network Lifetime

**Figure 4:**
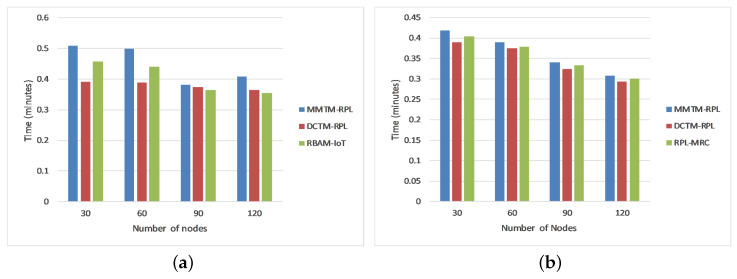

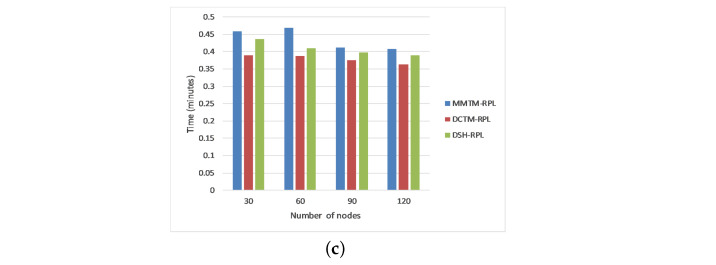
Comparison of network lifetime for different attacks: (**a**) Rank attack, (**b**) Sybil attack, and (**c**) Sinkhole attack.

Figure 4 depicts the average network lifetime in minutes for rank, Sybil, and sinkhole attacks. The simulation was run four times with 30, 60, 90, or 120 nodes. We compared the proposed mechanism (MMTM-RPL) with DCTM-RPL [28], RBAM-IoT [29], RPL-MRC [30], and DSH-RPL [25]. The number of nodes is shown on the *x*-axis, and the network lifetime is shown in minutes on the *y*-axis. For the different numbers of nodes, the network has a longer lifetime with the proposed mechanism than with the other mechanisms. For a rank attack, for example, the average network lifetimes were 0.45, 0.38, and 0.40 min for MMTM-RPL, DCTM-IoT, and RBAM-IoT, respectively. The clustering route optimization also played a crucial role. Overall, the overhead is shared by two layers (e.g., fog and sink). With MMTM-RPL, the message overhead is decreased by using MAs, which reduce the number of redundant messages received and forwarded by intermediate nodes. Thus, less energy is consumed, increasing the residual energy and ultimately increasing the network lifetime. In contrast, the message overhead is not managed effectively by the other mechanisms. In addition, note that the lifetime was higher for sinkhole and Sybil attacks.

#### 4.1.2. Average Residual Energy

**Figure 5:**
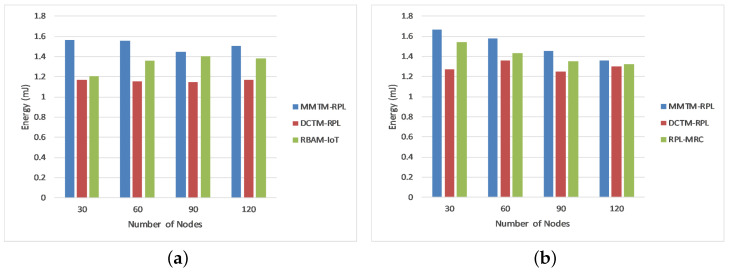

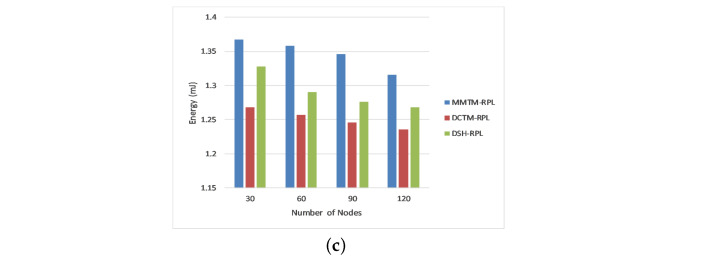
Comparison of average residual energy for different attacks: (**a**) Rank attack, (**b**) Sybil attack, and (**c**) Sinkhole attack.

Figure 5 shows the average residual energy for 30, 60, 90, or 120 nodes in millijoules. Again, MMTM-RPL is compared with DCTM-RPL [28], RBAM-IoT [29], RPL-MRC [30], and DSH-RPL [25]. The number of nodes is shown on the *x*-axis and the average residual energy is shown on the *y*-axis for the three types of attack.

Observe that our mechanism has a higher average residual energy for the different numbers of nodes. For a rank attack, the average residual energies were 1.52, 1.16, and 1.34 mJ for MMTM-RPL, DCTM-IoT, and RBAM-IoT, respectively. The main reason is that all calculations of trust parameters and trust values are handled by the fog layer rather than the nodes. Thus, the overhead for the nodes is minimized by transferring the work to the control layer. Nodes gather only messages and parameters and send them to the control layer. In contrast, for the other techniques, all computations were performed by the node layer. In addition, note that the average residual energy is a maximum for sinkhole and Sybil attacks.

The OF also directly influences the overall energy consumption of the network. Although the other mechanisms spend less energy than MMTM-RPL initially, over time, MMTM-RPL consumes less energy overall. Note that the trust computation considers contextual information and quality of service and influences network survivability and node failure. For peer-to-peer connections and identifying rogue nodes, the suggested technique has a longer network lifetime and lower energy consumption.

#### 4.1.3. Control Message Overhead

**Figure 6:**
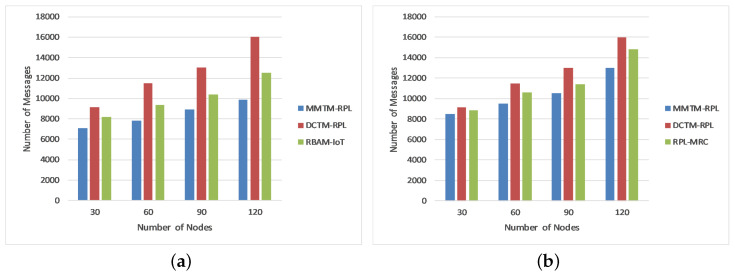

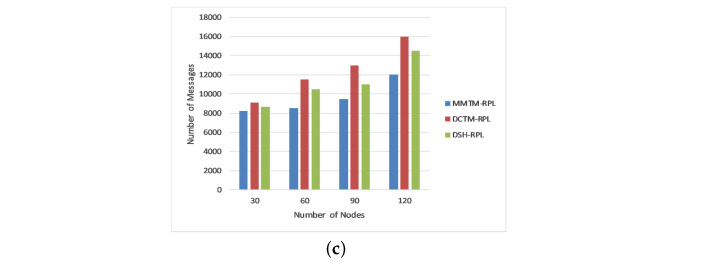
Comparison of control message overhead for different attacks: (**a**) Rank attack, (**b**) Sybil attack, and (**c**) Sinkhole attack.

Figure 6 shows the impact of varying the number of malicious nodes on the number of control messages exchanged in the network. As mentioned earlier, the ratio of malicious and benign nodes is 1:100; i.e., we introduced malicious nodes chronologically as 3, 6, 9, and 12 in the network of 30, 60, 90, and 120 nodes. Again, MMTM-RPL is compared with DCTM-RPL [28], RBAM-IoT [29], RPL-MRC [30], and DSH-RPL [25]. The number of nodes is shown on the *x*-axis, and the number of messages is shown on the *y*-axis for the three types of attack. Note that the message overhead was a minimum for MMTM-RPL. For a rank attack, the average numbers of messages were 8437.5, 12,410, and 10,125 for MMTM-RPL, DCTM-IoT, and RBAM-IoT, respectively. The control message overhead increased as the number of attackers increased because the attackers were scattered equally over the network. As a result, each attacker affects a huge number of valid nodes. For each mechanism, all nodes within the radio range of a malicious node reset their trickle timers whenever they receive a DIS multicast, causing them to transmit numerous DIO signals that are disseminated across the network. However, MMTM-RPL limits the number of trickle timer resets and the transmission of DIO messages to decrease network overhead. Compared to the state-of-the-art, the overhead was reduced by 39%, 48.7%, and 45% for three, six, or nine attackers, respectively. The average number of messages was a minimum for sinkhole and Sybil attacks. Figure 6 shows that the control message overhead grew as the number of data packets transmitted during the simulation increased. However, compared to other mechanisms, MMTM-RPL considerably minimized the control overhead.

#### 4.1.4. Attack Detection Rate

**Figure 7:**
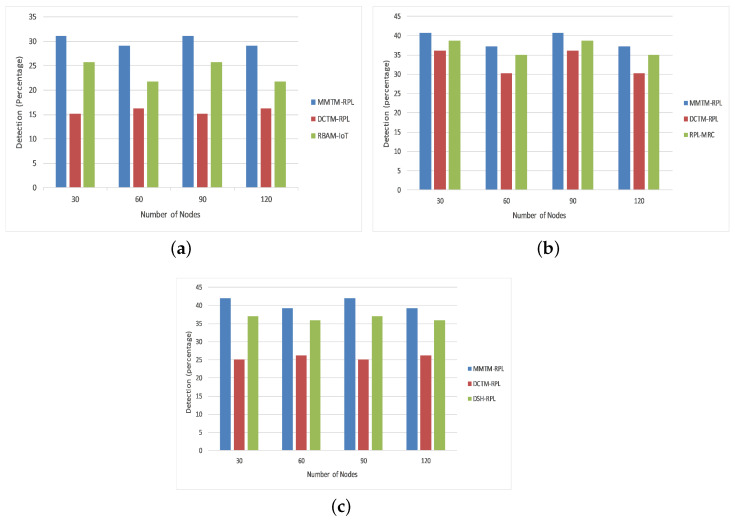
Comparison of attack detection rate for different attacks: (**a**) Rank attack, (**b**) Sybil attack, and (**c**) Sinkhole attack.

Figure 7 depicts the attack detection rate as a percentage of the total number of attacks. The simulation was run four times for 30, 60, 90, or 120 nodes. Our proposed mechanism was again compared with DCTM-RPL [28], RBAM-IoT [29], RPL-MRC [30], and DSH-RPL [25]. The numbers of nodes are shown on the *x*-axis and the attack detection rate is shown on the *y*-axis. The results are presented for rank, Sybil, and sinkhole attacks. Observe that the proposed mechanism has a higher attack detection rate for the different numbers of nodes. For a rank attack, the average detection rates were 0.28, 0.16, and 0.21 for MMTM-RPL, DCTM-IoT, and RBAM-IoT, respectively. A high detection rate means that attacks are detected earlier. MMTM-RPL has a higher detection rate since, because of the clustering, the parameters are collected faster. Moreover, the MAs played an important role here by sending parameters to the fog layer and by responding to attackers immediately. The attack detection rate was higher for sinkhole and Sybil attacks.

#### 4.1.5. Attack Detection Time

**Figure 8:**
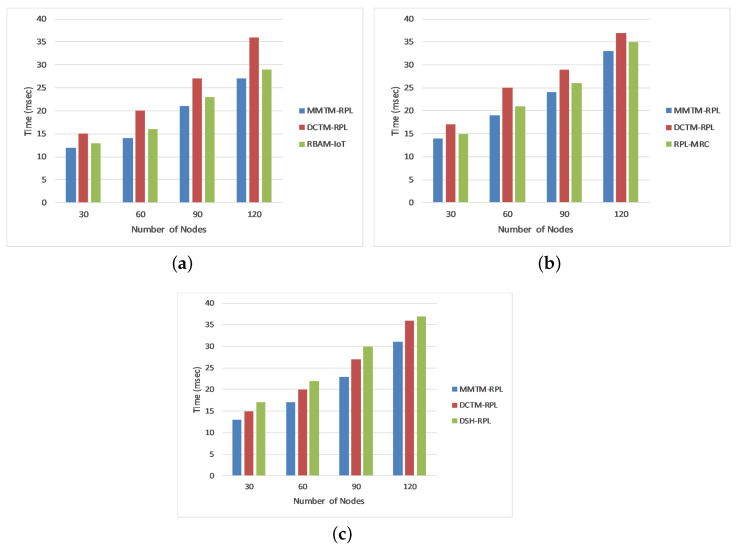
Comparison of attack detection time for different attacks: (**a**) Rank attack, (**b**) Sybil attack, and (**c**) Sinkhole attack.

The attack detection time is the average time taken to detect attackers in the network. It is calculated as the difference between the time when an attack was identified and the time when traffic began. We compared our proposed mechanism (MMTM-RPL) with DCTM-RPL [28], RBAM-IoT [29], RPL-MRC [30], and DSH-RPL [25]. The results are shown in Figure 8 for the three types of attack. The numbers of nodes are shown on the *x*-axis and the attack detection time is shown in milliseconds on the *y*-axis. Figure 8 shows that for a rank attack, the attack detection times were 18.50, 24.50, and 20.25 ms for MMTM-RPL, DCTM-IoT, and RBAM-IoT, respectively. Note that for MMTM-RPL, the time is a minimum. Similarly, it is lowest for sinkhole and Sybil attacks. This is because in MMTM-RPL, the clustering algorithm responds swiftly to the arrival and departure of nodes in the mobile networks. Secondly, the use of MAs also makes the proposed MMTM-RPL efficient [52], since they use dynamic itineraries. Thirdly, as the control message overhead in MMTM-RPL is low compared to the state of the art, the network performance plays an important role in decreasing the detection time.

#### 4.1.6. End-to-End Delay

**Figure 9:**
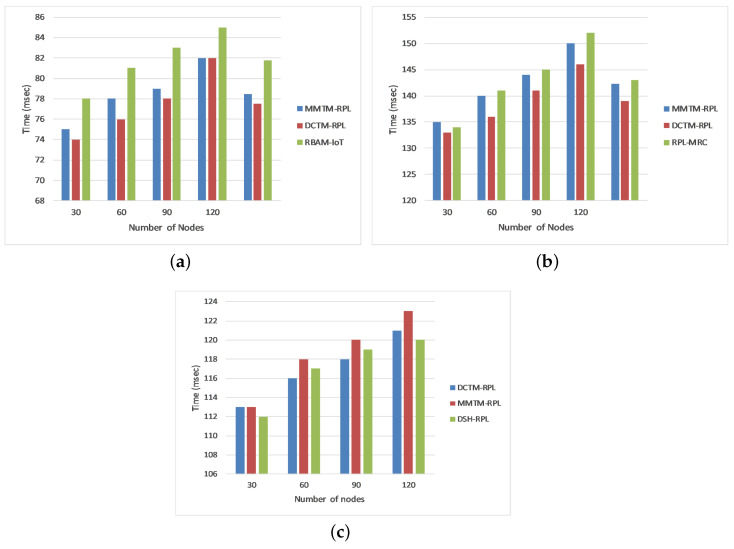
Comparison of end-to-end delay for different attacks: (**a**) Rank attack, (**b**) Sybil attack, and (**c**) Sinkhole attack.

The end-to-end delay is the duration between when packet transmission is initiated in the fog layer and its arrival at the DAG root in the node layer. Our proposed mechanism is compared again with DCTM-RPL [28], RBAM-IoT [29], RPL-MRC [30], and DSH-RPL [25]. The results are shown in Figure 9 for rank, Sybil, and sinkhole attacks. The numbers of nodes are shown on the *x*-axis, and the end-to-end delay in milliseconds is shown on the *y*-axis. The average end-to-end delays during a rank assault for DCTM-RPL, MMTM-RPL, and RBAM-IoT were 77, 78, and 81 ms, respectively. During a Sybil assault, the average end-to-end latency for DCTM-RPL, MMTM-RPL, and RPL-MRC was around 139, 142, and 143 ms, respectively. The end-to-end delays during sinkhole attacks for DCTM-RPL, MMTM-RPL, and DSH-RPL were around 117, 118, and 177 ms, respectively. The simulation findings reveal that the additional security mechanisms incorporated into an RPL-based LLN induce high delay. Moreover, since MMTM-RPL has an upper fog layer, it has slightly more latency than the other techniques, despite having a lower hop count. This indicates that the shortest path may not guarantee that delays and latency are minimized. Moreover, the trust calculation and propagation in the other approaches are done by the node layer, which reduces the delay.

## 5. Discussion

**Figure 10:**
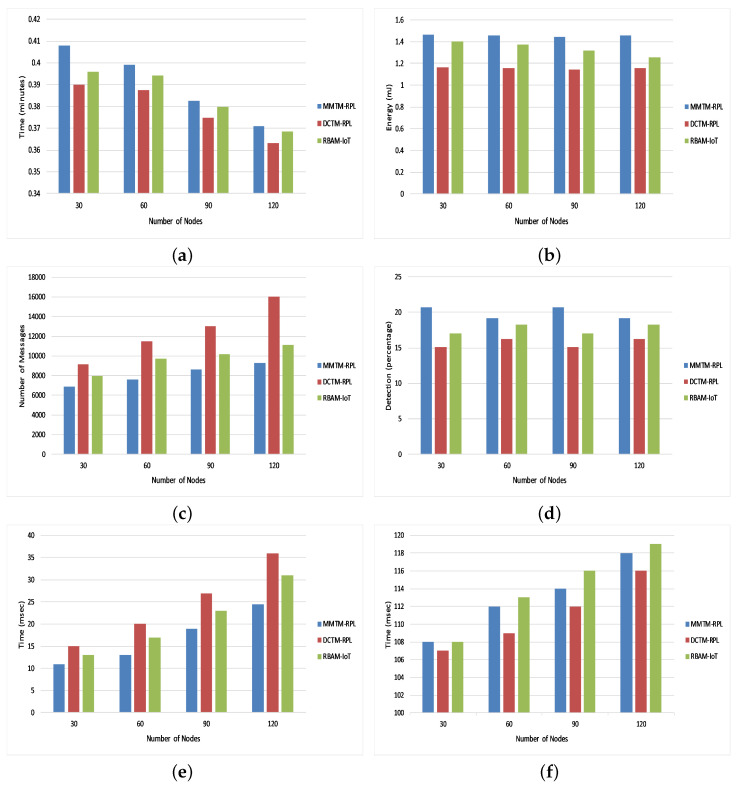
Overview of results for the different frameworks: (**a**) Network lifetime, (**b**) Average residual energy, (**c**) Control message overhead, (**d**) Attack detection rate, (**e**) Attack detection time, (**f**) End-to-end delay.

Figure 10a shows the average network lifetime under all three types of attack. As above, we compared the proposed mechanism (MMTM-RPL) with DCTM-RPL [28] and RBAM-IoT [29]. It is to be noted that the overall results are compared with only those that are state of the art, which cover most of the attacks. The results demonstrate that the network lifetime was higher for MMTM-RPL. Network lifetime depends on various network parameters. The key features of our mechanism, namely clustering, the layer-based architecture, and the division of tasks, reduce the computational overhead for each node. First, as mentioned above, energy at the node level is conserved by performing all calculations at the fog layer. Second, since the MAs collect the trust-related data from all clusters, the energy consumed when receiving and forwarding packets by the multi-hop network is substantially reduced. Thus, the network consumes less energy at the node level. There is more average residual energy at the node and network levels, and the lifetime of each node is extended, as is the network lifetime.

Figure 10b shows the average residual energy for all three types of attack. It confirms that MMTM-RPL has a higher average residual energy. Note that the overall residual energy was 25.84% higher for MMTM-RPL than for DTCM-RPL and 8.99% higher than for RBAM-IoT. This is mainly due to the layer-based architecture. Figure 10c shows the average number of control messages under all three types of attack. As the number of data packets transferred rises, so does the control message overhead. By using multiple MAs and efficient dynamic itineraries, MMTM-RPL greatly minimizes the control overhead, regardless of the data rate. The results show that there is 33.44% lower control message overhead with MMTM-RPL than with DCTM-RPL and 16.70% lower than with RBAM-IoT. Similarly, Figure 10d shows the average attack detection rate under all three types of attack. The overall improvement was 19.33% compared with DCTM-RPL and 8.17% compared with RBAM-IoT. Figure 10e gives the average attack detection time under all three types of internal attacks. There was a 30.81% and 19.32% improvement with the proposed MMTM-RPL compared with DCTM-RPL and RBAM-IoT, respectively.

Finally, Figure 10f shows the average end-to-end delay under all three types of attack. It was slightly higher for the proposed framework. On average, delays were 1.8% and 0.86% longer with MMTM-RPL than with DCTM-RPL or RBAM-IoT, respectively. The longer delays are due to the multi-hops in the network and because the trust calculations have been transferred to the fog layer. An agent has to fetch the trust parameters from the fog layer. For the other frameworks, trust is calculated by the device layer, which reduces the end-to-end delay. However, the increase in the delay for MMTM-RPL is negligible and can be ignored if the overall network lifetime and performance are better.

### Answers to Research Questions

Next, we provide answers to the RQs:

RQ1. How can security in RPL be improved to mitigate internal attacks while maintaining the mobility of the network?
Ans: In resource-constrained IoT devices, RPL plays an important role in communication and message passing. Nevertheless, it needs to be improved to provide security against potential internal attacks. Furthermore, in delay-sensitive IoT infrastructures, the timely and accurate detection of internal attacks is challenging. Therefore, MMTM-RPL enhances the efficiency and effectiveness of internal attack detection to improve the security of RPL-based frameworks, as shown in Figure 10a,d. The overall attack detection time is shorter compared to state-of-the-art approaches, as shown in Figure 10e. This is achieved by shipping complex computations to the fog layer to conserve the limited memory, energy, and computational resources. Although the end-to-end delay is slightly higher, the increase is negligible, as shown in Figure 10f.
RQ2. How can we ensure that data are gathered securely to protect RPL-based networks against internal attacks?
Ans: The literature review indicated that most state-of-the-art approaches do not consider network mobility, which is an essential feature of LLNs with IoT devices. Apart from minimizing the message, energy, and computational overheads, it is equally important to ensure data are gathered securely by the RPL infrastructure. Most LLN-based infrastructures use wireless communications for exchanging data, which are vulnerable to certain types of breaches [18]. In a trust-based mechanism, trust-related data (trust parameters) are important and must be secured. The integrity of trust is very much based upon the integrity of the trust parameters. Therefore, by authenticating the MAs sent to collect trust parameters, MMTM-RPL ensures that data are gathered securely so that trust values are effective and reliable. Therefore, it meets the mobile and dynamic requirements of RPL-based networks while securing them against internal attacks. This is achieved by generating new itineraries and initiating new MAs whenever a topology change occurs. Thus, the trust is calculated after each change as well as at fixed time intervals.
RQ3. In support of RQ1, can we minimize network congestion (overhead due to the number of messages exchanged) to improve network lifetime and minimize energy, memory, and computational overheads at the device level in RPL-based infrastructures?
Ans: In RPL-based IoT infrastructures, the data packets are sent in a multi-hop fashion. Due to the limited buffer size and bandwidth, exchanging massive amounts of data may increase network congestion and data-packet loss. To inoculate the network against internal attacks, apart from the data generated and exchanged by nodes, trust parameters are exchanged for trust assessment using the same channels. This increases the underlying channel load and also the data-packet loss rate and latency. However, the scope of this study is to minimize trust-related data congestion to support RPL only. To the best of our knowledge, in RPL-based trust mechanisms, trust-related data are either exchanged among nodes or routed toward the central entity in a multi-hop fashion, increasing the message overhead. Therefore, there is a dire need to minimize the amount of multi-hop trust-related data that is gathered, which MMTM-RPL achieves with MAs. These collect trust-related data from each node listed in their itineraries and give the data to the control layer. In this way, compared to state-of-the-art frameworks, the message overhead is reduced, as depicted in Figure 10c, and the energy is conserved at the node level, as shown in Figure 10b.

## 6. Conclusions

For LLNs, RPL is a promising routing protocol. However, security has not received sufficient consideration. In addition, LLN devices have limited memory, processing power, bandwidth, and energy resources, making RPL-based networks more vulnerable to various attacks. Internal attacks are hard to mitigate, as the attackers are genuine member nodes of the network. This paper presented a novel resource-efficient trust-based mechanism to extenuate rank, Sybil, and sinkhole attacks. It is a security framework that encompasses various mechanisms to enhance resource efficiency. It uses MAs with dynamic itineraries to collect trust parameters. To ensure that the itineraries are efficient, clusters are built using cluster trees. In addition, SL is used in the trust calculation. In summary, all calculations are performed in an upper layer, i.e., a fog layer. Our results suggest that the proposed framework performs better than state-of-the-art approaches. We intend to validate our proposed mechanism with artificial intelligence models. For this purpose, a personalized dataset will be constructed using simulation. As a part of this work, the dataset will be generated and validated. Subsequently, machine and deep learning models will be used to detect and isolate internal attacks. 

## Figures and Tables

**Table 1:** Comparison of the previously published studies in the state-of-the-art.

Ref.	Year	Attack Addressed	Mobility	Network Performance	Network Lifetime	Overhead		
						Energy	Message	Computation
[5]	2020	Sybil	×	Low	Short	🗸	🗸	×
[18]	2019	Rank, Sybil	×	Low	Short	🗸	🗸	🗸
[14]	2020	Rank, blackhole	×	Low	Short	🗸	🗸	🗸
[21]	2021	Blackhole, rank, sinkhole, man-in-the-middle	×	Low	Short	🗸	🗸	🗸
[22]	2021	Rank	×	Low	Short	🗸	🗸	🗸
[23]	2020	Sinkhole	×	Low	Short	🗸	🗸	×
[24]	2019	Rank, Sybil	×	Low	Short	🗸	🗸	🗸
[25]	2021	Sinkhole	×	Low	Long	×	🗸	🗸
[26]	2020	Sinkhole, rank	×	Low	Short	🗸	🗸	🗸
[27]	2017	Sybil	🗸	Low	Long	×	🗸	🗸
[28]	2019	Blackhole, rank, Sybil	🗸	Low	Long	×	🗸	🗸

**Table 2:** Simulation parameters.

Parameter	Value
Simulation tool	Contiki OS based Cooja 3.0
*MAC*	CSMA/CA+ MICMAC
Transport protocol	IPv6
Topology	Random
Node type	Tmote Sky
Simulation coverage area	100 m × 100 m
No. of nodes	30–120
No. of malicious nodes	3–12
Legitimate to malicious node ratio	1–100
$R_{x}$ ratio	30–100%
$T_{x}$ ratio	100%
$T_{x}$ range	50 m
Interference range	50 m
Traffic type rate	Constant bit rate of 6 pkt/min
Packet size	46 bytes
Routing protocol	RPL
Network protocol	IP based
Start delay	5 s
Simulation time	30–60 min
Mobility speed	0–6.23 km/h
Link failure model	UDG with distance

## Data Availability

Not applicable.
